# Avoidant/restrictive food intake disorder complicated by superior mesenteric artery syndrome in an adolescent male with Klinefelter syndrome: A case report

**DOI:** 10.1002/pcn5.70356

**Published:** 2026-06-08

**Authors:** Kohei Kamikawa, Ryohei Takada, Yuya Honda, Harue Goto, Takashi Okada

**Affiliations:** ^1^ Department of Psychiatry Nara Prefecture General Medical Centre Nara Japan; ^2^ Department of Psychiatry Nara Medical University School of Medicine Kashihara Japan

**Keywords:** autism spectrum disorder, avoidant/restrictive food intake disorder, Klinefelter syndrome, sensory sensitivity, superior mesenteric artery syndrome

## Abstract

**Background:**

Avoidant/restrictive food intake disorder (ARFID) is characterized by restrictive or avoidant eating not driven by weight or shape concerns and is often associated with autism spectrum disorder (ASD). Superior mesenteric artery (SMA) syndrome may occur in ARFID with significant weight loss; however, overlapping gastrointestinal symptoms may obscure its recognition.

**Case Presentation:**

A 16‐year‐old boy with a history of ASD and Klinefelter syndrome (47,XXY) presented with postprandial nausea, abdominal pain, fatigue, and rapid weight loss. He had chronic sensory hypersensitivity–related selective eating since early childhood and maintained a slender body habitus. Upon admission, his height and weight were 182 cm and 40.7 kg, respectively (body mass index [BMI] = 12.3). He had no drive for thinness, fear of weight gain, or body image disturbance and was diagnosed with ARFID. Abdominal ultrasonography revealed marked narrowing of the SMA–aorta angle (6.8°) and distance (3 mm), with duodenal compression, consistent with SMA syndrome. Enteral nutrition was initiated and gradually increased, accompanied by psychoeducation, meal support tailored to sensory sensitivities, and behavioral interventions. He showed weight restoration and improvement in gastrointestinal symptoms and duodenal obstruction, allowing a gradual transition to oral intake. Upon discharge, his weight was 49.0 kg (BMI = 14.8). Follow‐up ultrasonography revealed partial improvement in the SMA–aorta angle.

**Conclusion:**

SMA syndrome should be considered in patients with ARFID who present with gastrointestinal symptoms and rapid weight loss. Klinefelter syndrome may increase vulnerability to SMA syndrome, complicating clinical recognition. Combining nutritional rehabilitation, medical evaluation, and psychosocial interventions is important for effective complex eating disorder management.

## BACKGROUND

Avoidant/restrictive food intake disorder (ARFID) is characterized by restrictive or avoidant food intake not driven by a desire for thinness, fear of weight gain, or body image disturbance. ARFID is primarily associated with atypical sensory processing, including both exteroceptive sensitivities (e.g., taste and texture) and interoceptive disturbances.^1^, ^2^ ARFID has a multifactorial etiology that involves a complex interaction of neurodevelopmental traits and psychological, physical, and environmental factors.^2^, ^3^, ^4^ Sensory hypersensitivity, a core feature frequently observed in autism spectrum disorder (ASD), is an established risk factor for ARFID.^3^, ^5^ Approximately 12.5% of individuals diagnosed with ARFID have comorbid ASD.^6^

Rapid weight loss and malnutrition associated with eating disorders are risk factors for superior mesenteric artery (SMA) syndrome.^7^, ^8^ SMA syndrome involves a reduction in retroperitoneal adipose tissue, leading to narrowing of the aortomesenteric angle and subsequent compression of the third duodenal portion. This can cause gastrointestinal symptoms, including postprandial abdominal pain, nausea, and early satiety.^9^ SMA syndrome may further worsen restrictive eating behaviors, which subsequently exacerbates the underlying pathophysiology of ARFID.

Klinefelter syndrome (KS) is a sex chromosome aneuploidy most commonly characterized by a 47,XXY karyotype, with an estimated prevalence of approximately 1 in 500–1000 males.^10^ Individuals with KS often exhibit distinctive physical features, including tall stature, relatively long limbs, and reduced muscle mass, and show an increased susceptibility to neurodevelopmental traits, including ASD.^10^, ^11^, ^12^

SMA syndrome has been reported following rapid weight loss in anorexia nervosa (AN).^7^, ^13^ Because SMA syndrome is fundamentally related to substantial weight loss, it may also occur in cases of ARFID with marked weight loss. However, such cases may be less readily recognized, as the gastrointestinal symptoms of SMA syndrome can overlap with those of restrictive eating disorders, complicating differential diagnosis.^8^, ^14^ To our knowledge, SMA syndrome in a patient with comorbid KS has not been reported.

## CASE PRESENTATION

The patient was a 16‐year‐old boy who had exhibited difficulties in social interaction, restricted interests, and sensory hypersensitivity since early childhood. Accordingly, he had been diagnosed with ASD. Furthermore, he had a confirmed diagnosis of KS (47,XXY) following genetic testing. He did not present significant intellectual developmental delay but sensory hypersensitivity–related selective eating and food avoidance from early childhood. Although he experienced mild social difficulties related to ASD traits, no major school maladjustment, bullying, or marked psychosocial stressors were identified during adolescence. He had consistently maintained a slender body habitus.

From the age of approximately 13 years, his height showed a marked increase. By the age of about 15 years, his avoidant and restrictive eating behaviors became more pronounced. At the age of 16 years, he developed postprandial nausea, fatigue, and abdominal pain, accompanied by rapid weight loss. Upon admission, his height was 182 cm, consistent with the tall stature commonly observed in KS. Further, his weight was 40.7 kg, which corresponded to a body mass index (BMI) of 12.3 and indicated severe underweight status.

A comprehensive psychiatric assessment did not reveal psychopathological features characteristic of AN, including a drive for thinness, fear of weight gain, or body image disturbance. No apparent psychosocial trigger for the onset or worsening of eating disturbance was identified. In addition, no prominent depressive, anxiety, obsessive–compulsive, psychotic, or neurodevelopmentally related behavioral symptoms that could substantially account for the eating disturbance were observed during the clinical course. Persistent sensory hypersensitivity–related selective eating and food avoidance were evident. Considering the clinical course and eating behavior characteristics, he was diagnosed with ARFID based on the Diagnostic and Statistical Manual of Mental Disorders, Fifth Edition, Text Revision criteria.

Given the high risk of refeeding syndrome and the patient's strong aversion to food intake due to gastrointestinal symptoms (postprandial nausea, fatigue, and abdominal pain), enteral nutrition was initiated at 600 kcal/day and gradually increased. Because the recent rapid weight loss may have resulted from potential comorbid physical conditions, abdominal ultrasonography was performed, revealing a reduction in the superior mesenteric artery–aorta (SMA–Ao) angle (6.8°) and distance (3 mm), as well as compression of the ascending duodenal portion. Because these were consistent with the postprandial gastrointestinal symptoms and could not be explained by other medical conditions, the patient was diagnosed with SMA syndrome.

The patient was continued on enteral nutrition. Both the patient and his family received psychoeducation, which focused on sensory hypersensitivity associated with ASD, physiological effects of low body weight and malnutrition, and stepwise treatment goals. The patient's body weight recovered to 44 kg. He showed improvement in duodenal obstruction and related symptoms. Accordingly, a plan was established to gradually transition from enteral nutrition to oral intake.

During this transition, the patient received individualized meal support with careful consideration of his sensory sensitivities. Meals were tailored with respect to food type, flavor, and texture, and portion sizes were gradually increased. Behavioral interventions were implemented to mitigate avoidant and restrictive eating behaviors, including stepwise exposure to new or previously avoided foods, with positive reinforcement—praise and rewards—for enhancing motivation upon successful intake. Moreover, the patient was provided with a food diary for recording the meal content and intake volume, which promoted progress visualization and daily achievement.

**Figure 1:**
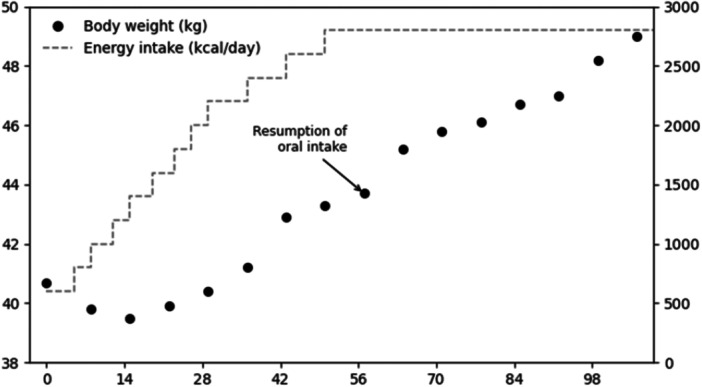
Clinical course during hospitalization. Body weight (black circles) was measured once weekly and plotted as discrete observations without interpolation. Prescribed daily energy intake (gray dashed line) was initiated at 600 kcal/day and increased twice weekly in 200‐kcal increments, reaching 2800 kcal/day. The horizontal axis represents the days since admission (Day 0). The left and right vertical axes indicate body weight (kg) and energy intake (kcal/day), respectively. Oral intake was resumed on Day 57, when body weight reached 43.7 kg, as indicated by the arrow.

Our multimodal approach combining nutritional therapy, psychoeducation for ARFID, meal support, and behavioral therapy increased the total caloric intake to 2800 kcal/day. He was discharged on hospital day 109 with a weight and BMI of 49.0 kg and 14.8, respectively. His clinical course is shown in Figure 1. At discharge, follow‐up abdominal ultrasonography performed in the supine position revealed improvement in the SMA–Ao angle and distance to 14.0° and 4 mm, respectively, with reduced compression of the ascending duodenum. Although these still indicated SMA syndrome, a marked improvement in postprandial gastrointestinal symptoms was evident. Additionally, while selective eating and food preferences persisted after discharge, they had a diminished impact on daily functioning.

This case report adheres to the CARE guidelines.

## DISCUSSION

We present a case of SMA syndrome precipitated by rapid weight loss in a patient with ARFID in the context of KS. The described clinical course highlights the importance of considering SMA syndrome in the differential diagnosis for patients with ARFID presenting gastrointestinal symptoms. Furthermore, it provides clinically meaningful insights into the contribution of KS‐related physical and neurodevelopmental characteristics to the development and pathophysiology of SMA syndrome.

Patients with AN, especially those experiencing rapid weight loss, have increased susceptibility to SMA syndrome.^7^, ^13^ In such cases, intensive nutritional rehabilitation has allowed weight gain, improvement in duodenal obstruction, alleviation of gastrointestinal symptoms, and increased oral intake.^15^ Notably, SMA syndrome may be overlooked given its symptomatic similarity to eating disorders.

In our case, we found marked narrowing of the SMA–Ao angle and shortening of the SMA–Ao distance, consistent with SMA syndrome and postprandial nausea, fatigue, and abdominal pain. These did not fully normalize but improved in parallel with an improvement in postprandial gastrointestinal symptoms, suggesting that radiological changes may not perfectly correlate with clinical recovery, especially in severe malnutrition.^16^, ^17^ Weight loss associated with ARFID may have precipitated SMA syndrome, with the resulting gastrointestinal symptoms further reinforcing restrictive eating behaviors and creating a self‐perpetuating vicious cycle.

ARFID is distinct from AN in that it lacks the psychopathological characteristics of a drive for thinness, fear of weight gain, or body image disturbance. Rather, it is driven by factors such as sensory hypersensitivity, aversion to specific foods, or fear of postprandial discomfort.^1^, ^2^ Consequently, weight loss and gastrointestinal symptoms in ARFID may be readily attributed to psychological or behavioral factors alone. Accordingly, there is a risk of overlooking comorbidities such as SMA syndrome. Therefore, comprehensive evaluation—including detailed history‐taking, physical examination, and abdominal imaging—is essential for accurate differential diagnosis in patients with ARFID who present gastrointestinal symptoms.

In KS, skeletal growth is promoted by a gene dosage effect of the SHOX gene associated with X chromosome polysomy, which predisposes affected individuals to tall stature.^18^, ^19^ Hypogonadism following puberty leads to delayed epiphyseal closure and relatively reduced muscle mass in the limbs, which yields characteristic physical features such as tall stature and disproportionately long extremities.^10^, ^19^ In taller individuals, changes in body weight may represent a smaller proportion of total body mass. Accordingly, the effects of weight loss may be distributed across a larger skeletal frame, which impedes visual detection of weight loss. During adolescence, a rapid increase in height may mask the rapid weight loss, delaying recognition of eating disorders or associated medical conditions such as SMA syndrome. In our case, the patient's slender body habitus from early childhood may have further obscured the recognition of clinically significant weight loss. Taken together, in patients with KS, weight loss may be underestimated based on appearance alone, which increases the risk of delayed intervention. In addition to these physical characteristics, adolescents with KS may experience psychosocial difficulties related to pubertal development, body image, self‐esteem, and social adaptation. Although no clear psychosocial precipitants or prominent psychiatric comorbidities were identified in our patient, such vulnerabilities may have indirectly influenced eating behaviors, illness recognition, or treatment engagement. Therefore, psychosocial assessment may also be important when evaluating eating disturbances in adolescents with KS.

In our case, although intensive nutritional therapy improved duodenal obstruction and gastrointestinal symptoms, there was relatively slow weight gain. In individuals with KS, pubertal testosterone deficiency impairs muscle protein synthesis and promotes muscle catabolism, which leads to insufficient increases in fat‐free mass, particularly skeletal muscle mass.^19^, ^20^ Accordingly, weight gain may be attenuated even with increased caloric intake. Moreover, although reduced skeletal muscle mass lowers basal metabolic rate, energy intake during malnutrition periods and refeeding is preferentially utilized for physiological recovery processes rather than being reflected as weight gain.^21^ Taken together, the relatively slow weight gain observed in our patient may have been influenced by the body composition and endocrine characteristics associated with KS.

Additionally, the physical phenotype associated with KS may have contributed to the development of SMA syndrome. Beyond the reduction in retroperitoneal adipose tissue, tall and slender body types may be predisposing factors for SMA syndrome.^22^, ^23^ Such body proportions may increase the relative distance and alter the spatial relationship between the SMA and duodenum, which results in a more acute aortomesenteric angle and facilitates duodenal compression. In our patient, the combination of KS‐related body habitus with ARFID‐related low body weight and malnutrition may have synergistically increased the vulnerability of SMA syndrome. However, it remains difficult to determine the precise timing of SMA syndrome onset. Moreover, the implied causal relationships among these factors must be interpreted cautiously given that they are based on retrospective inference.

Our findings suggest that nutritional management for malnutrition is effective in both ARFID and SMA syndrome. Notably, while prioritizing intensive nutritional therapy to address SMA syndrome, a parallel multimodal approach was implemented, which incorporated general psychoeducation, meal support tailored to sensory sensitivities, and behavioral interventions. It is unlikely that adequate therapeutic benefit would have been achieved with any single intervention alone; notably, partial resolution of SMA syndrome appeared to be a key prerequisite for the subsequent success of psychological and behavioral interventions.

This case report has several limitations. First, since this is a single case report, caution is warranted in generalizing the observed associations between eating behavior abnormalities and SMA syndrome in patients with KS. Second, it is difficult to precisely establish the temporal relationship between changes in eating behavior and the onset of SMA syndrome, which limits definitive conclusions regarding causality. This is further impeded by the lack of long‐term follow‐up. Third, in patients with KS, the evaluation of treatment response should ideally extend beyond body weight and BMI to include multidimensional assessments of body composition, particularly fat‐free mass and skeletal muscle mass. In the present case, skeletal muscle mass was not assessed, which may have limited the comprehensive evaluation of treatment effects. Fourth, endocrine parameters, including serum testosterone levels, were not systematically assessed; therefore, the potential contribution of hypogonadism to the patient's physical and psychiatric symptoms could not be fully evaluated, and testosterone replacement therapy was not formally considered during the treatment course. In addition, hypogonadism associated with KS may also have influenced fatigue, motivation, and psychological functioning, which could have indirectly affected eating behavior, treatment engagement, and recovery from malnutrition. Future studies accumulating similar cases are warranted to systematically examine how the physical and neurodevelopmental characteristics associated with KS interact with medical comorbidities to influence disease mechanisms and treatment responsiveness.

## CONCLUSION

This article describes a case of SMA syndrome precipitated by rapid weight loss associated with ARFID in a 16‐year‐old boy with KS. This case report has three important clinical implications. First, in patients with significant weight loss, including those with ARFID, gastrointestinal symptoms and weight loss should not be solely attributed to psychological or behavioral factors; instead, it is important to consider comorbid medical conditions, including SMA syndrome, in the differential diagnosis. Second, the interaction of KS‐related physical characteristics—such as tall stature and relatively long limbs—with ARFID‐related low body weight and malnutrition may increase vulnerability to the development of SMA syndrome. Third, improvement of SMA syndrome may require not only intensive nutritional rehabilitation but also a multimodal treatment approach that integrates meal support and behavioral interventions tailored to ASD‐related sensory sensitivities. Taken together, these findings highlight the importance of comprehensive medical and psychosocial assessment as well as individualized, multidisciplinary intervention in complex eating disorder presentations.

## AUTHOR CONTRIBUTIONS

Kohei Kamikawa, Ryohei Takada, Yuya Honda, and Harue Goto treated the patient at our hospital. Kohei Kamikawa, Ryohei Takada, Yuya Honda, and Harue Goto treated the patient in the outpatient clinic. Kohei Kamikawa, Ryohei Takada, Yuya Honda, Harue Goto, and Takashi Okada wrote the manuscript. All authors participated in the discussion, writing, and revisions, and read and approved the final version of the manuscript.

## CONFLICT OF INTEREST STATEMENT

The authors declare no conflicts of interest.

## ETHICS APPROVAL STATEMENT

According to the institutional policy, ethical approval was not required for this case report.

## PATIENT CONSENT STATEMENT

Written informed consent for publication of this case report was obtained from the patient and his parent. The consent forms are securely retained by the authors.

## CLINICAL TRIAL REGISTRATION

N/A.

## Data Availability

All data supporting the findings of this case report are included in the main text of the article. No additional datasets were generated or analyzed during the preparation of this report.
